# Effect of air-polishing on surface roughness of composite dental restorative material – comparison of three different air-polishing powders

**DOI:** 10.1186/s12903-020-1007-y

**Published:** 2020-01-30

**Authors:** Joanna Janiszewska-Olszowska, Agnieszka Drozdzik, Katarzyna Tandecka, Katarzyna Grocholewicz

**Affiliations:** 1 Department of Interdisciplinary Dentistry Pomeranian Medical, University in Szczecin, al. Powstancow Wlkp, 72, 70-111 Szczecin, Poland; 2 Faculty of Mechanical Engineering Koszalin, University of Technology, ul, Raclawicka 15-17, 75-620 Koszalin, Poland

**Keywords:** Sodium bicarbonate, Glycine, Erythritol, Prophylaxis, Filling

## Abstract

**Background:**

Increased composite roughness enhances bacterial adhesion and discoloration, thus increasing the risk of gingival inflammation and secondary caries. Concerns about detrimental effects of sodium bicarbonate on surface roughness influenced the development of less abrasive powders: a glycine-based powder and an erythritol-based powder, additionally - sodium bicarbonate-based powder of reduced grain size. However, there is limited evidence on effects of these materials on the surface of dental fillings. The aim of the present study was to compare the effects of three air-polishing powders (of a reduced abrasiveness) on surface roughness of microhybrid restorative composite material.

**Material and methods:**

Microhybrid light-cure resin composite samples were placed on 64 plaster cubes and light-cured through polyester strips. Surface roughness was measured using laser confocal microscope (magnification 2160x). The specimens were randomly divided into three groups (*n* = 20, 20 and 24) and air-polished with: sodium bicarbonate (40 μm), glycine (25 μm) and erythritol (14 μm), respectively. Then surface roughness was remeasured, keeping the same field of observation. Specialized 3D analysis software was used for data processing. Parameters according to ISO 25178: Sa, Sq, Sku, Sp, Sv, Sz, Ssk were used to describe surface roughness.

**Results:**

Sa, Sq, Sp, Sv, Sz increased significantly following air polishing. Ssk was significantly higher, whereas Sku was significantly lower in sodium bicarbonate and erythritol groups than before air polishing. Comparison between the three powders revealed that Sa was significantly higher in sodium bicarbonate group than in glycine group. Sku was significantly higher in glycine and erythritol groups than in sodium bicarbonate group.

**Conclusions:**

Sodium bicarbonate has a stronger detrimental effect on composite surface than glycine or erythritol. No advantage of erythritol comparing to glycine could be found.

## Introduction

Hygiene maintenance therapy is a crucial part of periodontal and restorative treatment [1–3]. Biofilm and tooth deposits are usually repeatedly removed at regular intervals, even in patients with low risk of periodontal diseases and caries development. There are several procedures for plaque and extrinsic stains removal, among them application of slurry of pressurized air, abrasive powder and water (air-polishing), as alternatives to conventional techniques. Air-polishing, as compared with the use of rubber cups, hand and ultrasonic scalers, is a highly effective, easy and rapid technique [4–8]. It causes less operator fatigue, and improves access to hardly accessible tooth surfaces [8].

The air polishing technology began with the application of sodium bicarbonate based air powders [8]. The conventional sodium bicarbonate powder, with particles up to 250 μm, is regarded as a high-level abrasive material, carrying a risk to soft and hard tissues as well as restorative materials [8–11]. Air-polishing composite resins with sodium bicarbonate results in a noticeable surface wear [12–16], that may entail both aesthetic and health aspects. Moreover, surface roughness increase occurs, which plays a crucial role in bacterial adhesion and biofilm formation, thus increasing the risk of gingival inflammation and secondary caries [17–20].

Concerns about harmful effects of sodium bicarbonate influenced the development of less abrasive powders [2]. In 2003 a new powder containing glycine and 10 years later, an erythritol-based powder were marketed [21, 22], additionally sodium bicarbonate-base powder with reduced granule-metric size up to 40 μm has become available. There is limited information about topical effects of these more recently launched materials. The available studies suggest significantly reduced abrasive effects on composite resins of glycine based than sodium bicarbonate based powders [15].

To the best of authors’ knowledge there are no studies evaluating effects of an erythritol powder on composite surface roughness.

The aim of this in vitro study was to compare three different air-polishing powders in terms of their effect on the surface roughness of microhybrid restorative composite material.

## Materials and methods

Microhybrid light-cure resin composite (Charisma, Heraeus Kulzer, Hanau, Germany) samples were placed on 64 plaster cubes, light-cured through polyester strips (Direkta Dental, Upplands Väsby, Sweden) for 40 s each using dental curing light (T-led, SternWeber, Poland) and numbered in sequence.

The surface roughness of all specimens was measured using a laser confocal microscope (Lext OLS4000, Olympus) with a 100x lens (MPLAPON100xLEXT), under the magnification 2160x. The field of observation and measurement was 128 μm × 128 μm. The confocal mode was used to analyse the height information, whereas laser microscope mode was used for sample observation and acquiring images. The measuring units in z-azis were 10 nm. Each sample was aligned according to x, y and z coordinates from the marked starting point using the motorized table of the microscope.

Then the specimens were randomly divided into three groups of 20, 20 and 24 specimens respectively and air-polished, using three different air-polishing powders:

1. Group SB - sodium bicarbonate – Air-Flow Supragingival Comfort Classic (EMS SA, Switzerland),
2. Group G - glycine – Air-Flow Subgingival Perio (EMS SA, Switzerland),
3. Group E - erythritol - Air-Flow Sub + Supragingival Plus (EMS SA, Switzerland).

The average grain sizes of the powders provided by the manufacturer were: 40 μm, 25 μm and 14 μm, respectively. The air-polishing procedure was performed by the same periodontal specialist using the standard unit (Air-Flow Master, EMS SA, Switzerland), according to the manufacturer recommended settings at the pressure of 2.5 bar and with three different powder chambers for each powder. In order to achieve reproducible working conditions powder chambers were refilled to the recommended maximum level after each air-polishing run. The efforts were made to duplicate clinical procedure as much as possible. The standard air polishing nozzle, designed for supragingival application, was used with spraying distance of 3 mm, at 45^o^ angle between nozzle and specimens surface with spraying time of 5 s counted down via electronic control of the device. During the application the nozzle was kept in a constantly sweeping movement as in clinical practice.

Then the surface roughness of each sample was remeasured, keeping the same field of observation. Specialized 3D analysis computer software - TalyMap Platinum (Taylorhobson Ltd., USA) was used for data processing, which comprised: surface levelling, non-measured points filling (using a smooth shape calculated from the neighbours) and shape (form) removal. The following height parameters according to ISO 25178 were used to describe composite surface roughness:

- Sa - arithmetical mean height of the surface,
- Sq - root mean square height of the surface,
- Sku – kurtosis of the surface
- Sp – maximum peak height
- Sv – maximum pit depth
- Sz - maximum height of the surface,
- Ssk - skewness of height distribution.
- Shapiro-Wilk test at the level α = 0.05 was used in order to check for data normality.

Analysis of variance (ANOVA) was used to compare between variables of normal distribution and for the latter - Kruskal-Wallis test. The roughness parameters were compared between the whole sample (*n* = 64) before air-polishing (C - control) and groups SB, G and E polished with three different powders. Then, surface roughness parameters were compared between the three groups polished with different powders. In order to verify the sample size sample size analysis was performed using an online calculator (powerandsamplesize.com). With the clinical significance of 0,25 μm for Sa and Sq, the sample size yielded 20 and 6, respectively.

## Results

Data normality has been presented in Table 1. Most data were characterized by a non-normal distribution.

**Table 1:** Analysis of data normality (Shapiro-Wilk test)

Variable	Before air-polishing	After air-polishing		
		Group SB	Group G	Group E
Sa	< 0,001*	0,885	0,085	0,116
Sq	< 0,001*	0,266	0,358	0,017*
Sku	< 0,001*	< 0,001*	0,001*	< 0,001*
Sp	< 0,001*	0,02*	0,165	< 0,001*
Sv	< 0,001*	0,041*	0,223	0,001*
Sz	< 0,001*	0,247	0,191	< 0,001*
Ssk	0,021*	0,487	0,181	0,003*

* *p* < 0.05 is evidence of non-normal distribution

No statistically significant differences (ANOVA) were found between the study groups before air-polishing, thus further comparisons were made between the whole sample before air-polishing (*n* = 64) and groups SB, G and E after air-polishing.

**Fig. 1:**
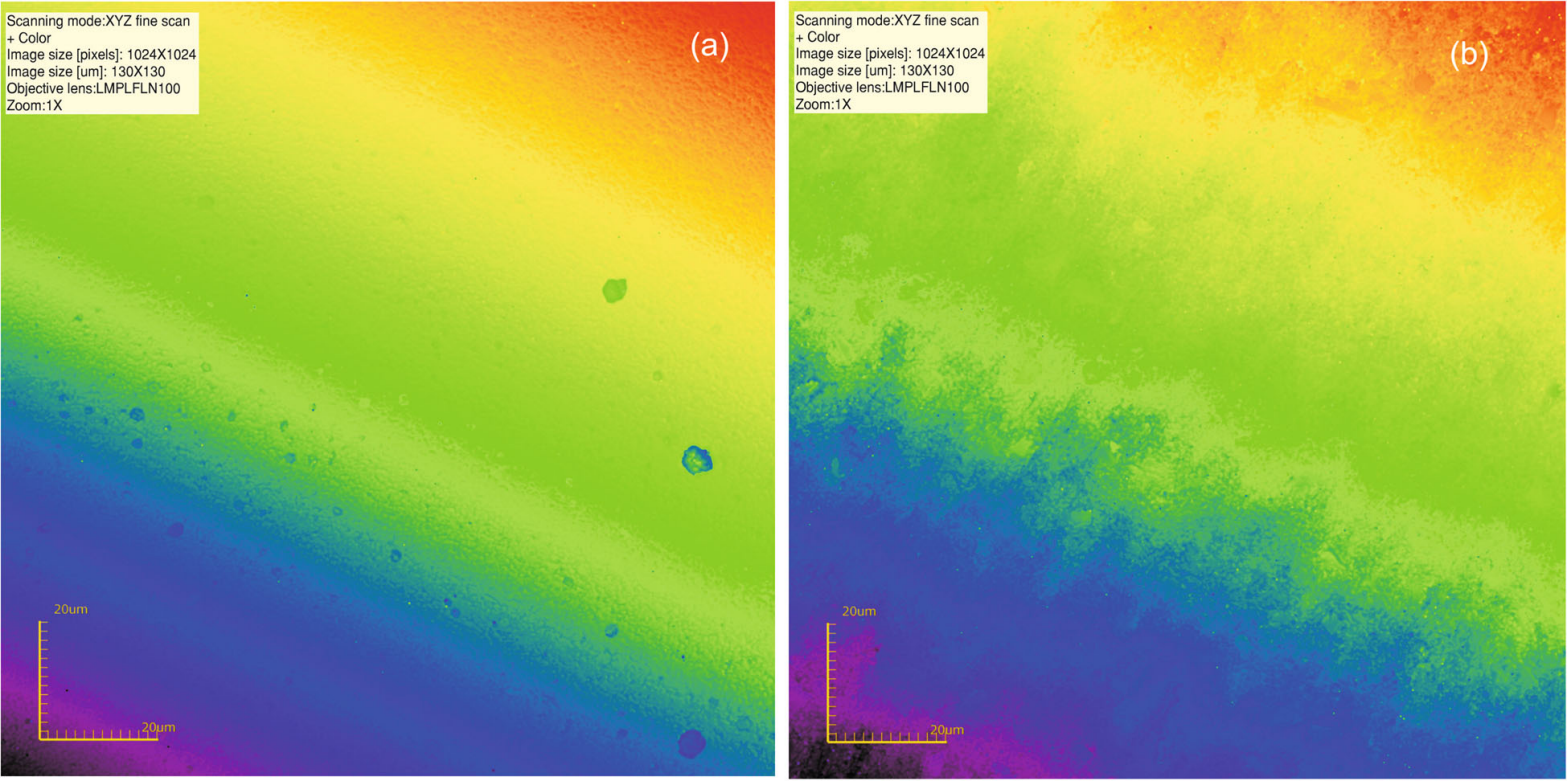
**a** Typical composite surface; **b** Following air-polishing with sodium bicarbonate

**Fig. 2:**
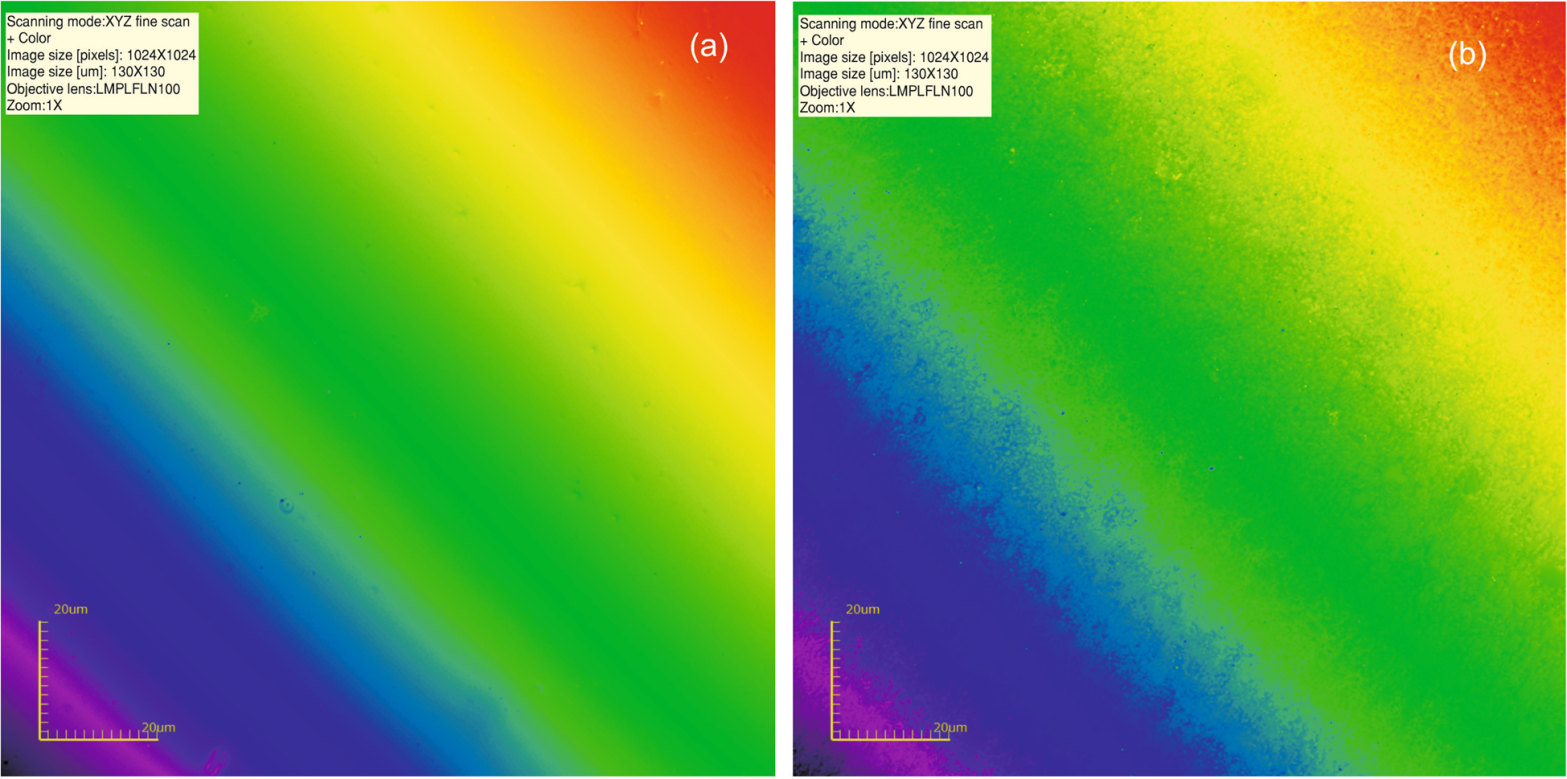
**a** Intact composite surface; **b** Following air-polishing with glycine

**Fig. 3:**
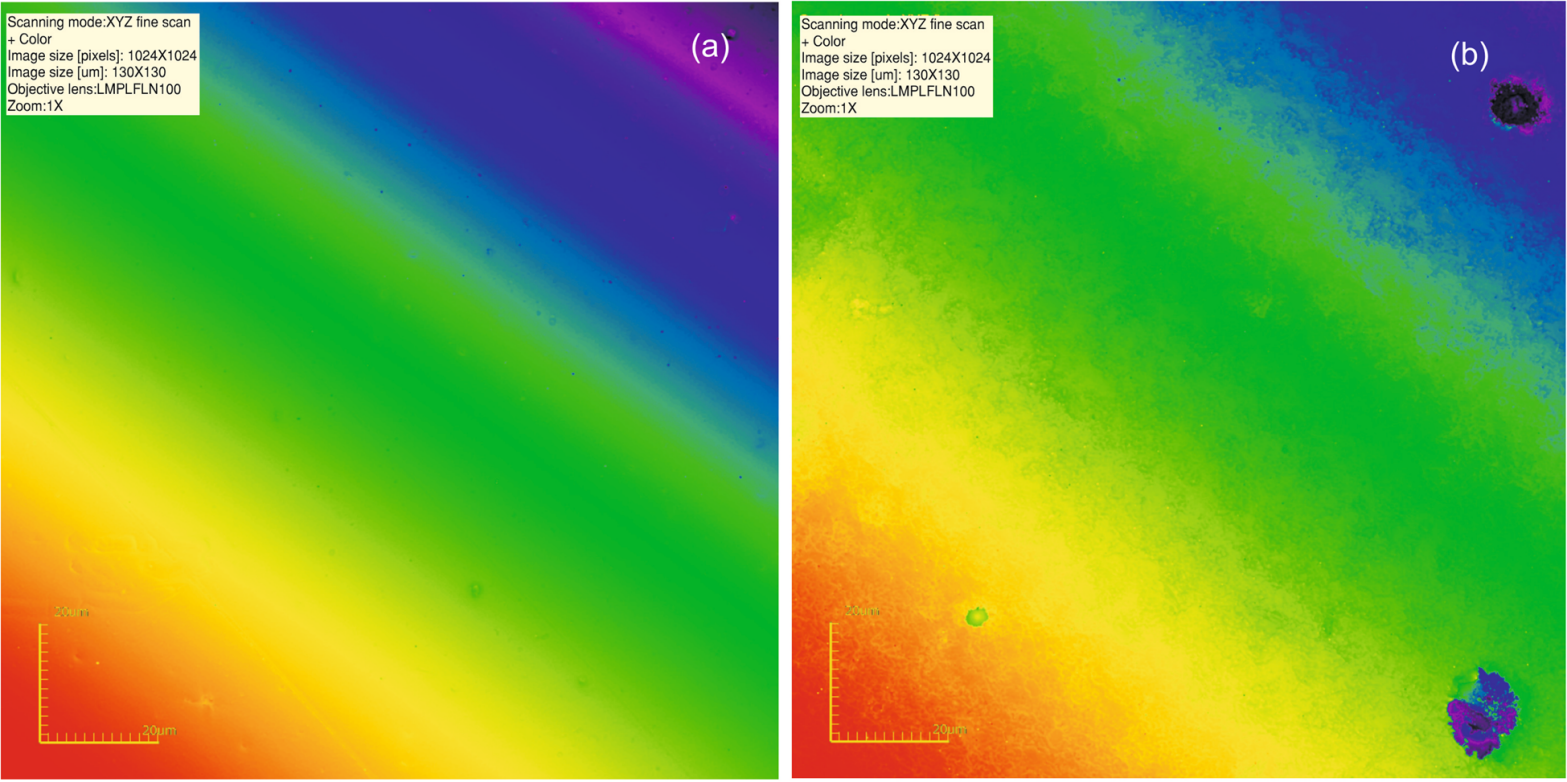
**a** Composite surface before air-polishing; **b** Following air-polishing with erythritol

The distribution of the roughness parameters before and after air-polishing is presented in Table 2, whereas raw data – as a Additional file 1. Typical composite surfaces from each group of specimen are presented in Figs. 1, 2 and 3. All variables compared were significantly different (*p* < 0.05) after air-polishing for all the groups analysed. A post-hoc analysis (Dunn test) proceeded proved that:

- Sa, Sq, Sp, Sv, Sz were significantly higher in groups SB, G and E than before air polishing,
- Ssk was significantly higher in groups SB and E than before air polishing,
- Sku was significantly lower in groups SB, G and E than before air polishing.

**Table 2:** Distribution of roughness parameters before and after air-polishing

Variable	Group	N	Mean	SD	Median	Min	Max	Q1	Q3	p *	p **
Sa [μm]	Before air-polishing (C)	64	0,03	0,02	0,02	0,01	0,1	0,02	0,03	< 0,001	
	Group SB	20	0,28	0,11	0,28	0,04	0,5	0,23	0,34		0,029
	Group G	20	0,18	0,11	0,21	0,03	0,36	0,09	0,27	SB,G,E > C	SB > G
	Group E	24	0,21	0,14	0,2	0,04	0,55	0,09	0,29		
Sq [μm]	Before air-polishing (C)	64	0,09	0,08	0,07	0,03	0,51	0,05	0,11	< 0,001	
	Group SB	20	0,46	0,23	0,41	0,06	0,96	0,31	0,56		0,14
	Group G	20	0,31	0,16	0,3	0,07	0,74	0,21	0,4	SB,G,E > C	
	Group E	24	0,38	0,26	0,34	0,08	1,06	0,23	0,44		
Sku	Before air-polishing (C)	64	439,95	565,46	238,05	4,04	3733,08	142,3	539,86	< 0,001	
	Group SB	20	31,17	52,85	13,79	3,99	244,69	7,7	30,19		0,016
	Group G	20	104,49	115,96	35,88	4,72	365,73	17,83	171,55	C > SB,G,E	G,E > SB
	Group E	24	132,18	262,67	73,26	6,6	1310,53	16,49	114,43		
Sp [μm]	Before air-polishing (C)	64	1,65	2,26	0,93	0,13	13,49	0,41	1,85	< 0,001	
	Group SB	20	3,75	2,7	3,45	0,56	11,9	2,14	4,82		0,775
	Group G	20	3,29	2,12	3,19	0,52	9,02	1,7	4,78	SB,G,E > C	
	Group E	24	3,99	3,18	3,53	0,22	15,83	1,76	5,28		
Sv [μm]	Before air-polishing (C)	64	3,39	2,9	2,73	0,83	17,68	1,47	4,08	< 0,001	
	Group SB	20	4,82	2,48	3,91	1,89	10,89	2,69	6,95		
	Group G	20	4,61	2,02	4,91	1,74	8,1	2,6	6,08	SB,G,E > C	0,798
	Group E	24	5,44	3,3	4,34	1,81	15,25	3,1	6,78		
Sz [μm]	Before air-polishing (C)	64	5,04	4,66	4,16	1,01	31,17	2,39	5,52	< 0,001	
	Group SB	20	8,58	4,28	7,97	2,48	19,07	5,5	10,65		
	Group G	20	7,9	3,12	6,56	3,15	13,84	6,02	10,27	SB,G,E > C	0,77
	Group E	24	9,43	5,58	8,56	3,64	31,08	6,31	10,4		
Ssk	Before air-polishing (C)	64	−8,89	12,05	−8,97	−47,86	24,53	−15,17	−2,68	< 0,001	
	Group SB	20	−0,94	3,06	−0,57	−8,98	5,06	−2,61	0,09		0,395
	Group G	20	−4,04	5,91	−1,87	−15,82	6,29	−8,16	0,48	SB,E > C	
	Group E	24	−3,26	6,62	−1,35	−25,23	4,72	−6,68	0,72		

* Kruskal-Wallis test + post- hoc analysis (Dunn test)

** ANOVA + post-hoc analysis (Fisher’s LSD test) for Sa, Kruskal-Wallis test + post-hoc analysis (Dunn test) for the latter variables

Comparing between the three powders, it has been found that:

- Sa was significantly higher in group SB than in group G
- Sku was significantly higher in groups G and E than in group SB.

## Discussion

The present in vitro study provides comprehensive information about the effects of three commercially available types of air-polishing powders, i.e. sodium bicarbonate-, glycine- and erythritol- based on the surface roughness of microhybrid composite resin used for dental restorations. All tested powders, although characterized by a reduced abrasiveness, caused a statistically significant increase in surface roughness. Various composite materials may have different resins and contain filler particles of different size and composition. Thus surface susceptibility to damage may differ. Moreover, it is known, that surface roughness is material dependent. Thus the purpose of this study was to assess and compare the effect of air-polishing powders on one composite resin material, which is currently used in a daily practice for anterior and posterior restorations.

The use of a confocal microscope has the advantage of objective quantitative measuring 3D roughness parameters of the surface. However, the surface alteration caused by air-polishing would be also visible in scanning electron microscopy.

The surface texture of composites has influence on plaque accumulation, discoloration and wear [23]. Decreased bacterial adhesion was observed at surface roughness of 0.15 μm [24]. Moreover, the tip of the tongue is able to detect a surface roughness change of 0.3 μm, thus a smooth surface adds to the patient’s comfort [25]. Furthermore, the final surface polish influences the aesthetics of composite restorations and contributes to the gloss meter [26] as well as a better color stability [27].

It is believed that smoothest surface is obtained when composite is polymerized against a matrix strip. Clinically, composite restorations may present a high variety of finishing and polishing quality. Moreover, through the time in the oral cavity bacterial esterases can degrade composites, thereby increasing the surface roughness of restorations [28, 29]. In the present study, smooth composite surfaces were produced by the use of matrix strips in order to obtain a uniformity of the initial roughness. However, it can be supposed, that clinically, the initial as well as the post-treatment surfaces could be much rougher.

Air-polishing composite resin could potentially influence its hardness. However, no studies concerning influence of air-polishing on composite resin nanohardness or wear properties could be found. It has been found that the smoothest composite surface obtained under a polyester matrix rich in organic resin is characterized by a lower hardness than following finishing procedures [30, 31]. Finishing procedures applied to different composites may produce surfaces of various roughness and hardness [31]. Understanding the effect of air-polishing on the microhardness of finished composite surface would require a thorough study.

Increased roughness is a sign of surface damage. Surface damage resulting from kinetic abrasion is influenced by the characteristics of the particles applied. The greater the particle size, hardness and angularity, the more abrasive the slurry [32].

The study evaluated powders based on sodium bicarbonate, glycine and erythritol, with a reduced grain size of 40 μm, 25 μm and 14 μm respectively. On the Mohs hardness scale the sodium bicarbonate rank 2.8 and glycine and erythritol 2 [33]. Reduction in micron size and hardness enabled safe removal of biofilm and stains above and below the gum line.

The fact that arithmetical mean height of the surface (Sa), root mean square height of the surface (Sq), maximum peak height (Sp), maximum pit depth (Sv) and maximum height of the surface (Sz) were higher in all three experimental groups than before air polishing reflects a significant degree of surface damage resulting from air-polishing.

The surface skewness represents the degree of symmetry of the surface heights about the mean plane. Ssk < 0 indicates the predominance of valley structures. Ssk had a negative value before air-polishing, which increased significantly in groups SB and E after air polishing. However, no statistically significant differences were found between the three powders.

Sku values above 3.0 indicate the presence in inordinately deep peaks or valleys. The fact that it was significantly reduced in all groups after air-polishing reflects a modification of the composite resin surface. Sku was significantly higher in groups G and E than in group SB, showing the highest surface damaging potential of bicarbonate comparing to the other powders tested. It seems that glicyne and erythritol have a similar surface-damaging potential. No other studies including erythritol powder could be found for comparison.

## Conclusion

It can be concluded that the sodium bicarbonate-based air-polishing powder has a stronger detrimental effect on composite surface in terms of its roughness compared to erythritol and glicyne. No advantage of erythritol-based powder comparing to glicyne could be found in this study.

## Supplementary information

**Additional file 1.** Roughness parameters.

## Data Availability

Raw data has been submitted as a supplemental file.
